# The *Theobroma cacao* B3 domain transcription factor *TcLEC2* plays a duel role in control of embryo development and maturation

**DOI:** 10.1186/1471-2229-14-106

**Published:** 2014-04-24

**Authors:** Yufan Zhang, Adam Clemens, Siela N Maximova, Mark J Guiltinan

**Affiliations:** 1 The Huck Institute of the Life Sciences, The Pennsylvania State University, 422 Life Sciences Building, University Park, PA 16802, USA; 2 The Department of Plant Science, The Pennsylvania State University, University Park, PA 16802, USA

**Keywords:** LEC2, Cacao zygotic embryo development, Cacao somatic embryogenesis, Embryogenic potential, Fatty acid biosynthesis

## Abstract

**Background:**

The *Arabidopsis thaliana LEC2* gene encodes a B3 domain transcription factor, which plays critical roles during both zygotic and somatic embryogenesis. LEC2 exerts significant impacts on determining embryogenic potential and various metabolic processes through a complicated genetic regulatory network.

**Results:**

An ortholog of the Arabidopsis Leafy Cotyledon 2 gene *(AtLEC2)* was characterized in *Theobroma* cacao (*TcLEC2). TcLEC2* encodes a B3 domain transcription factor preferentially expressed during early and late zygotic embryo development. The expression of *TcLEC2* was higher in dedifferentiated cells competent for somatic embryogenesis (embryogenic calli), compared to non-embryogenic calli. Transient overexpression of *TcLEC2* in immature zygotic embryos resulted in changes in gene expression profiles and fatty acid composition. Ectopic expression of *TcLEC2* in cacao leaves changed the expression levels of several seed related genes. The overexpression of *TcLEC2* in cacao explants greatly increased the frequency of regeneration of stably transformed somatic embryos. *TcLEC2* overexpressing cotyledon explants exhibited a very high level of embryogenic competency and when cultured on hormone free medium, exhibited an iterative embryogenic chain-reaction.

**Conclusions:**

Our study revealed essential roles of TcLEC2 during both zygotic and somatic embryo development. Collectively, our evidence supports the conclusion that *TcLEC2* is a functional ortholog of *AtLEC2* and that it is involved in similar genetic regulatory networks during cacao somatic embryogenesis. To our knowledge, this is the first detailed report of the functional analysis of a *LEC2* ortholog in a species other then Arabidopsis. *TcLEC2* could potentially be used as a biomarker for the improvement of the SE process and screen for elite varieties in cacao germplasm.

## Background

The tropical tree *Theobroma cacao* L. is cultivated as a cash crop in many developing countries and provides the main ingredients for chocolate production. In 2011, the global market value of the chocolate industry surpassed $100 billion and the demand for cacao beans (seeds) continues to increase [1]. Cacao trees are generally highly heterozygous and when propagated by seed, only a small fraction of individuals are high producing [2-4]. Thus, vegetative propagation systems provide a means to avoid the issue of trait variation, through cloning of the top elite individual genotypes.

Several methods of vegetative propagation are commonly used with cocoa (grafting and rooted cuttings techniques). In addition, *in vitro* somatic embryogenesis (SE) tissue culture offers an approach to speed up the development and deployment of genetically improved genotypes because of its potentially very high multiplication rate and scalability. Protocols for primary and secondary SE in cacao have been well documented [5-8]. However, SE can be limited by embryogenic efficiency, which varies significantly between genotypes. A deeper understanding of the genes and mechanisms involved in regulating the SE process in cacao could potentially lead to improvement of SE methods for commercial plant production. To characterize the mechanisms regulating embryogenesis, we have chosen a translational biology approach, leveraging the knowledge gained from the model plant Arabidopsis.

In Arabidopsis, leafy cotyledon (*LEC*) transcription factors, including *AtLEC1 *[9], *AtLEC2 *[10] and *AtFUS3 *[11] have been characterized as master regulators of zygotic embryo development [12]. The *AtLEC2* gene encodes a B3 domain transcription factor, which binds specifically to the *RY* motifs in the 5′ flanking regions of *AtLEC2*-induced genes [13]. *AtLEC2* is exclusively expressed in developing zygotic embryos during both the early development and maturation phases. It is required for development and maintenance of suspensors and cotyledons and for the acquisition of desiccation tolerance and inhibition of premature germination [10]. Loss-of-function Arabidopsis *lec2* mutants exhibit pleiotropic effects including abnormal suspensor anatomy, abnormal cotyledons with trichomes, precociously activated shoot apical meristems, highly pigmented cotyledon tips with prominent anthocyanin accumulation and reduced accumulation of seed storage compounds [10,14,15]. AtLEC2 functions both by inducing a cascade effect of other transcription factors controlling various developmental and metabolic pathways as well as through direct targeting and regulation of seed storage genes [16,17]. For example, *AtWRI1*, another key transcription factor crucial to embryo development, is a direct target of *AtLEC2* and is necessary to regulate normal fatty acid biosynthesis [17].

*LEC* genes are also important during somatic embryogenesis. For example, *lec2* mutants produced SEs in Arabidopsis at a very low efficiency [18], while ectopic expression of *AtLEC2* in Arabidopsis and tobacco vegetative tissue induced SE formations [10,19,20]. In addition, the capacity for SE was abolished in double (*lec1 lec2, lec1 fus3, lec2 fus3*) or triple (*fus3 lec1 lec2*) *LEC* mutants, which further confirms the critical and redundant roles of LEC proteins during SE [18]. It is well known that exogenous application of hormones, such as synthetic auxin (2,4-D) and cytokinin, are required to induce SE [21-23] and furthermore, a functional interaction between auxin and *AtLEC2* has been observed. In Arabidopsis, the expression of *AtLEC2* was significantly up-regulated in response to exogenously applied 2,4-D during the induction phase of SE [14]. Also, expression levels of *AtLEC2* were observed to be significantly higher in embryogenic callus compared to the non-embryogenic callus of the same age [14]. Interestingly, overexpression of *AtLEC2* in immature zygotic embryo transgenic explants was able to induce direct somatic embryogenesis, with little callus formation and in the absence of exogenous auxin [14]. Regarding this, Stone and Wojcikowska proposed that *AtLEC2* may activate genes involved in auxin biosynthesis, such as *YUC1*, *YUC2*, *YUC4 and YUC10 *[24,25]. Taken together, *AtLEC2* is essential for maintaining embryogenic competency of plant somatic cells through complex interactions with transcriptional regulators and auxin [26].

The *LEC* genes are also involved in regulation of fatty acid biosynthesis and storage lipid deposition during embryo development. The seed specific overexpression of *ZmLEC1* and *BnLEC1* led to 35% and 20% increase in seed oil contents in maize and canola, respectively [27,28]. Ectopic expression of *AtLEC2* in Arabidopsis leaves resulted in the accumulation of seed specific fatty acids (C20:0 and C20:1) and increased the mRNA level of oleosin [16]. Furthermore, a direct downstream target of *AtLEC2*, *AtWRI1* is known to control fatty acid metabolism through interactions with key genes upstream in the pathway [29].

Although the functions of *AtLEC2* have been extensively studied in Arabidopsis, and homologs described in several plant species [30], a functional ortholog has not been characterized in any other plants to date. We present here the identification of a putative ortholog of *AtLEC2* in cacao, *TcLEC2*. We characterized the expression patterns of *TcLEC2* during both zygotic and somatic embryogenesis and explored the relationships between the activity of TcLEC2 in modulating the embryogenic potential of callus and in regulation of the fatty acid biosynthesis pathway.

## Results

### Gene isolation and sequence comparison

The Arabidopsis *AtLEC2* gene (At1G28300) is part of a large family of B3 domain containing proteins involved in a wide variety of functions. In the Arabidopsis genome, 87 genes were previously annotated as B3 domain containing genes that were further classified into five different families: auxin response factor (ARF), abscisic acid-insensitive3 (ABI3) of which *AtLEC2* is a member, high level expression of sugar inducible (HSI), related to ABI3/VP1 (RAV) and reproductive meristem (REM) [31].

In order to identify a putative ortholog of *AtLEC2* in cacao, the full-length amino acid sequence of Arabidopsis *AtLEC2* was blasted against the predicted proteome of the Belizean Criollo genotype (B97-61/B2) (http://cocoagendb.cirad.fr/ [32]) using blastp algorithm with E-value cut-off of 1e^-5 ^[33], which resulted in identification of 13 possible candidate genes (Additional file 1). As a second approach to identify cacao *LEC2* gene (s), the predicted protein sequences of each of the 13 candidate genes were used to search the predicted proteome from a second sequenced cacao genome of cv. Matina 1–6 v1.1 (http://www.cacaogenomedb.org [34]) by Blastp and a set of nearly identical cognate genes were identified for each (Additional file 1). No additional related genes were identified in this variety of cacao. Of the 13 candidate genes, the gene *Tc06g015590* resulted in the best alignment with *AtLEC2*, resulting in a blastp expect value of 3E-75.

**Figure 1:**
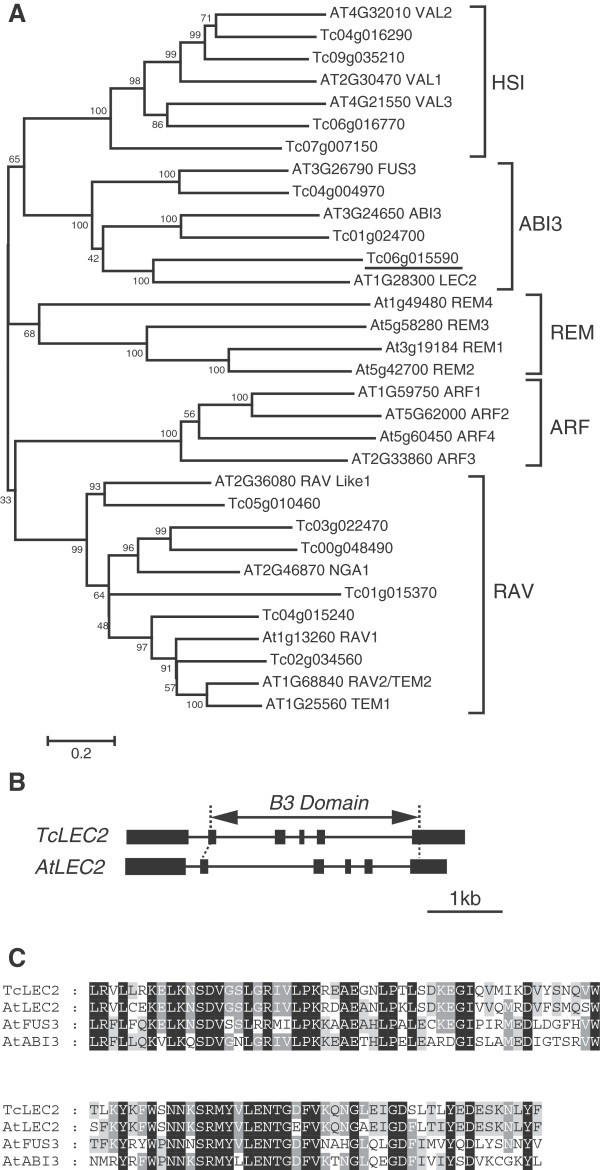
**Phylogenetic analysis and gene structure of B3 domain containing genes in cacao. A**. Unrooted neighbor-joining consensus tree of full-length amino acid sequences of selected Arabidopsis and *Theobroma cacao* B3 domain containing genes. The scale bar represents 0.2 estimated substitutions per residue and values next to nodes indicate bootstrap values from 1000 replicates. Five families of B3 domain containing genes were identified. The gene most closely related to *AtLEC2* (underlined) was designated as *TcLEC2*. **B**. Comparison of *TcLEC2* and *AtLEC2* gene structures. Boxes represent exons and lines indicate introns. Location of the conserved B3 domain is indicated. **C**. Amino acid alignment of B3 domains from *TcLEC2*, *AtLEC2*, *AtFUS3*, and *AtABI3*. Residues in black boxes are identical in all four proteins; residues in dark grey boxes are identical in three of four proteins; residues in light grey boxes are identical in two of four proteins.

To identify the most likely *AtLEC2*-orthologous gene, a phylogenetic analysis was performed with the 13 candidate cacao genes and several representative genes from each of the five B3 domain families in Arabidopsis (Figure 1A). The 13 cacao genes clustered with three of the B3 domain containing gene families (HIS, ABI3, RAV). Three cacao genes clustered within the ABI3 subfamily, with one cacao gene pairing with each of the three Arabidopsis members of this group (*Tc04g004970* with *AtFUS3*, *Tc01g024700* with *AtABI3* and *Tc06g015590* with *AtLEC2*), again suggesting that *Tc06g015590* is the most likely ortholog of *AtLEC2* in the cacao genome. This gene exists as a single copy and we tentatively designated it as *TcLEC2*.

The annotation of *TcLEC2* (*Tc06g015590*) in the cacao genome database predicted two translational start sites 72 bp apart. PCR primers were designed based on the most 5′ potential translation start site and a predicted full-length coding sequence of *TcLEC2* was amplified from cDNA extracted from *SCA6* mature zygotic cotyledons. A 1368 bp fragment was sequenced and after alignment with the *TcLEC2* genomic sequence, a gene model was constructed, consisting of six exons and five introns, nearly identical to the *AtLEC2* gene structure (Figure 1B). The lengths of the first and last exons differ slightly and the remaining four are identical. The *TcLEC2* encodes an open reading frame of 455 amino acid residues with the B3 domain predicted in the central region of the polypeptide. The full-length TcLEC2 protein shares 42% identity with AtLEC2 (Additional file 2); however, they are 81% identical within the B3 domain (Figure 1C).

### *TcLEC2* is expressed primarily in endosperm and early mature embryo cotyledons

**Figure 2:**
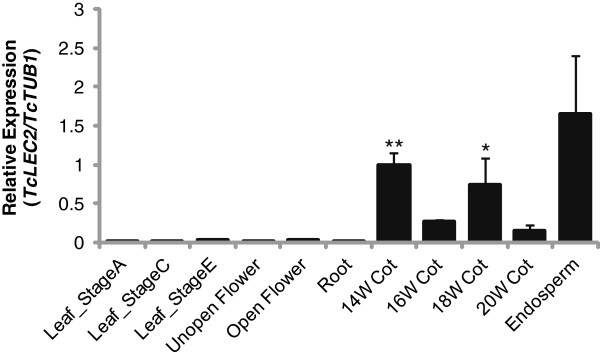
**TcLEC2 expression pattern in different cacao tissues.** Tissues include: leaves and flowers at different developmental stages, roots and zygotic cotyledons from seeds collected at 14, 16, 18 and 20 weeks after pollination (14 W Cot, 16 W Cot, 18 W Cot and to 20 W Cot respectively). The expression levels were analyzed by qRT-PCR and *TcLEC2* gene normalized relative to that of *TcTUB1* gene. Bars represent mean values (n = 3; mean ± SE). Significance was established by t-test (**represents p-value < 0.01by t-test; *represents p-value < 0.05 by t-test).

To investigate the function of TcLEC2 in cacao, its expression was measured by qRT-PCR in various tissues including: leaves at developmental stages A, C, and E (defined in [35]), unopened flowers, open flowers, roots, endosperm and zygotic seeds at 14, 16, 18, 20 weeks after pollination (WAP). A cacao beta-tubulin gene (*TcTUB1*, *Tc06g000360*) that was previously shown to exhibit stable expression levels during cacao seed development (unpublished data) was used for normalization. *TcLEC2* was exclusively expressed in cacao endosperm and cotyledon (Figure 2), and significant levels of transcript were not detected in other tissues, consistent with the *AtLEC2* expression pattern in Arabidopsis [10,30]. Moreover, the expression of *TcLEC2* was significantly higher in cacao cotyledons at 14 and 18 WAP compared to 16 and 20 WAP, stages previously defined as the onsets of cacao embryo morphogenesis and the seed maturation phase, respectively [36]. A similar biphasic expression pattern was reported for *LEC2* in Arabidopsis [30], suggesting a potential role of TcLEC2 in early developmental induction and in maturation phases of zygotic embryogenesis. Notably, the transcript of *TcLEC2* was accumulated to high levels in endosperm (90 days after pollination) when the embryo had just begun development (Figure 2). The endosperm functions to provide nutritive support to the developing embryo, and for crosstalk between maternal tissue and the embryo, being a critical determinant of successful embryo development [37]. Therefore, the abundance of *TcLEC2* transcript in the endosperm of developing cacao ovules suggests that *TcLEC2* expression in endosperm could be involved in controlling embryo initiation in cacao.

### Ectopic expression of *TcLEC2* was sufficient to activate seed specific gene expression in cacao leaves

**Figure 3:**
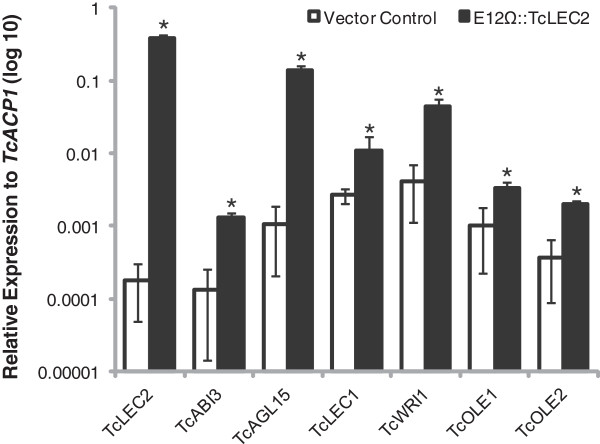
**Genes induced by ectopic overexpression of *TcLEC2 *in attached cacao leaf transient assay.** Expression levels of *TcLEC2* and TcLEC2 induced genes (*TcAGL15*, *TcABI3*, *TcWRI1*, *TcOLE1*, and *TcOLE2*) in *TcLEC2* ectopic expressing attached cacao leaves compared to vector control by qRT-PCR. The expression levels of genes were normalized relative to that of *TcACP1*. (n = 3, mean ± SE) *represents for p-value < 0.05 by t-test.

To test the function of cacao TcLEC2 in regulation of gene expression and to identify its putative downstream targets, a rapid transient transformation assay using cacao leaf tissue was utilized [38] (see Methods, Additional file 3). *TcLEC2* was ectopically overexpressed under the *E12-Ω* modified CaMV35S promoter (E12Ω::TcLEC2*,* pGZ12.0108, GenBank Accession: KF963132, Additional file 4) in fully expanded young stage C cacao leaves using *Agrobacterium* vacuum infiltration. *Agrobacterium* containing empty based vector pGH00.0126 (control vector, GenBank Accession: KF018690, EGFP only) was also infiltrated in parallel as a control. As expected, *TcLEC2* was highly expressed only in leaves transformed with E12Ω::TcLEC2 vector but was not detectable in control leaves (Figure 3). To identify the potential targets of TcLEC2, a set of cacao putative orthologs of genes involved in seed development in Arabidopsis was also assayed via qRT-PCR (Table 1). The predicted ortholog of *AGAMOUS-Like* 15, a MADS box type transcription factor involved in the induction of somatic embryogenesis from shoot apical meristems [39], was highly induced (>129 fold) by *TcLEC2* ectopic overexpression (Figure 3), which was consistent with the observation that LEC2 and AGL15 were able to activate each other in Arabidopsis [40]. The predicted ortholog *of ABA INSENSITIVE 3* (*ABI3*), which encodes a B3 domain transcription factor active during seed development and previously identified as a downstream target of AtLEC2 in Arabidopsis [41,42], was also induced (>9 fold) by TcLEC2 (Figure 3). However, another B3 domain transcription factor *FUSCA 3* (*FUS3*) [43,44] was not responsive to *TcLEC2* overexpression in leaf tissues under our experimental conditions (Table 1). The predicted ortholog *of WRINKLED 1* (*WRI1*), an AP2/EREB family transcription factor that is the direct downstream target of AtLEC2 and specifies AtLEC2 function toward fatty acid biosynthesis pathway in Arabidopsis [17,29], was induced more than ten-fold by TcLEC2 (Figure 3). Moreover, two genes encoding for *OLEOSIN* proteins, involved in the structure of oil bodies, were also activated in cacao leaves by *TcLEC2* ectopic overexpression (Figure 3). Collectively, these results indicated that TcLEC2 was sufficient to induce the ectopic transcription of several important seed specific genes in cacao leaves, supporting its function as a key regulator of embryo and seed development.

**Table 1:** **Changes in gene expression levels in response to *TcLEC2 *** **ectopic expression in leaf tissues of genes involved in various dimensions of seed development calculated from qRT-PCR measurements**

**Gene**	**Gene_ID**	**Vector control**	**E12Ω::TcLEC2**	**Fold change**	**Gene function**
*TcAGL15*	*Tc01g040120*	0.0002 ± 0.0001	0.3821 ± 0.0422	129.2	*MADS* box transcription factor, regulate GA biosynthesis
*TcWRI1*	*Tc10g012790*	0.0001 ± 0.0001	0.0013 ± 0.002	10.53	*AP2/ERWEBP* transcription factor, regulate fatty acid biosynthesis and embryo development
*TcABI3*	*Tc01g024700*	0.001 ± 0.0008	0.1355 ± 0.0103	9.57	*B2, B3* domain transcription factor, regulation ABA induced gene expression
*TcOLE2*	*Tc09g004410*	0.0027 ± 0.0006	0.011 ± 0.0061	5.49	Oleosin, oily body structure protein
*TcLEC1*	*Tc07g001180*	0.0041 ± 0.003	0.433 ± 0.0133	4.14	*HAP3* subunit CCAAT-binding transcription factor
*TcOLE1*	*Tc04g001560*	0.001 ± 0.0008	0.0033 ± 0.0006	3.27	Oleosin, oily body structure protein
*TcKASII*	*Tc09g006480*	0.0004 ± 0.0003	0.002 ± 0.0002	-2.77	3-ketoacyl-ACP-synthase II
*TcFUS3*	*Tc04g004970*	nd	nd	Not Induced	*B3* domain transcription factor, direct bind to RY motif
*TcLEC1_Like*	*Tc06g020950*	0.0011 ± 0.0006	0.0016 ± 0.0003	Not Induced	*HAP3* subunit CCAAT-binding transcription factor
*TcVicilin*	*Tc04g024090*	0.0003 ± 0.0002	0.0002 ± 0.0001	Not Induced	most abundant seed storage protein in cacao
*TcBBM*	*Tc05g019690*	nd	nd	Not Induced	*AP2* transcription factor in developing embryos and seeds
*TcPKL*	*Tc09g001610*	0.3973 ± 0.0578	0.3928 ± 0.0733	Not Induced	*CHD* chromatin remodeling factor
*TcWUS*	*Tc01g001780*	nd	nd	Not Induced	Master regulator of stem cell fate determination in shoot apical meristem
*TcYUC2*	*Tc09g009820*	0.0016 ± 0.0002	0.001 ± 0.0001	Not Induced	flavin monooxygenase in auxin biosynthesis
*TcYUC4*	*Tc09g013260*	0.0093 ± 0.0069	0.0085 ± 0.0021	Not Induced	flavin monooxygenase in auxin biosynthesis

Expression levels were normalized to *TcACP1* (column 3 and 4). Fold changes were calculated as a ratio of expression levels induced by TcLEC2 relative to expression levels in tissues transformed with the vector control lacking *TcLEC2* and are the mean of three biological replicates (n = 3, mean ± SE). Significance was established by t-test with p-value cut-off of 0.05.

### *TcLEC2* expression is associated with embryogenic competency of callus cells

**Figure 4:**
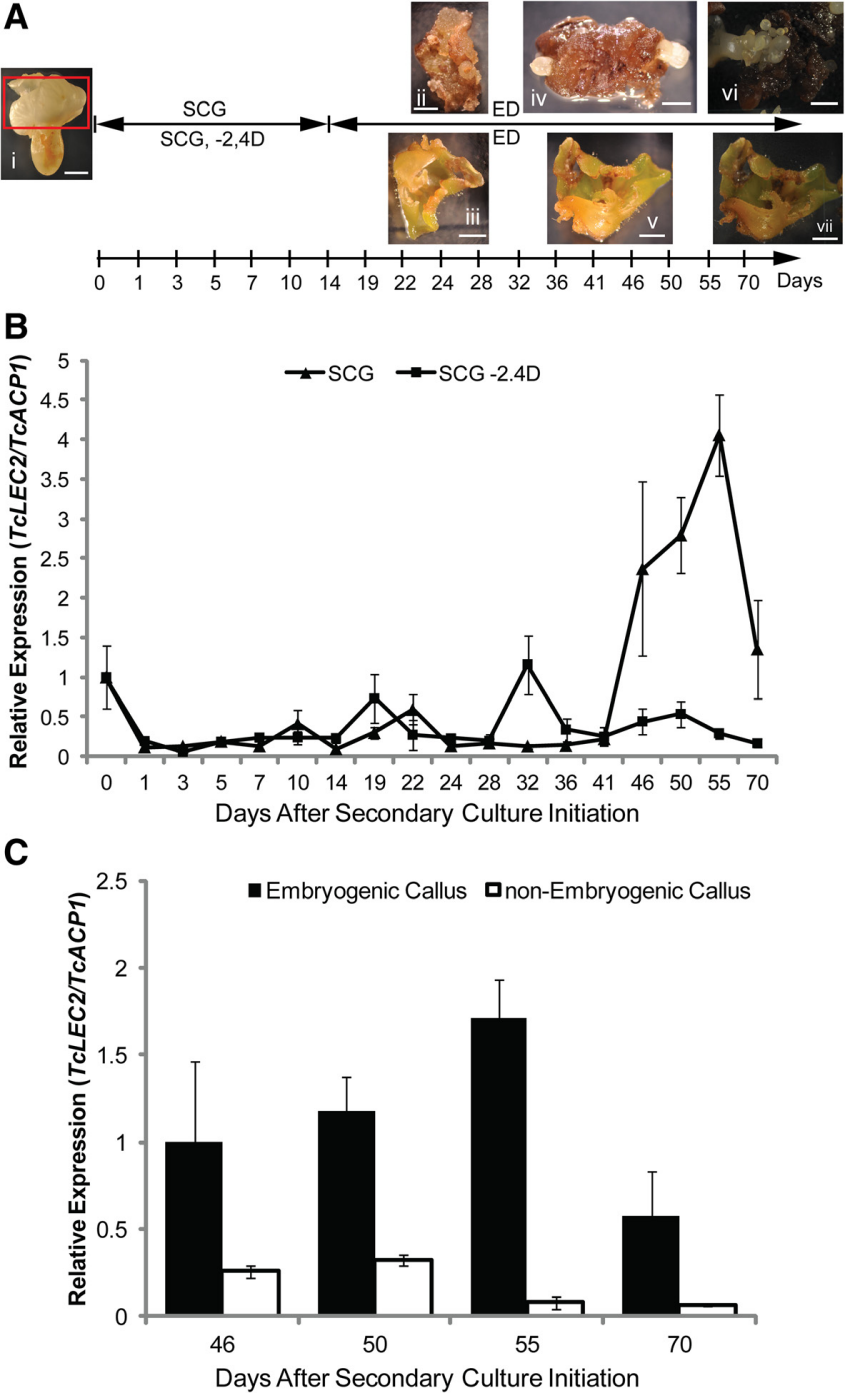
***TcLEC2 *expression correlates with embryogenic potential. A**. Illustration of the cacao secondary somatic embryogenesis stages and time frame, indicating the points used for sample collections. Representative images of several key stages of embryo development: (i) cotyledon stage *PSU-SCA6* embryo used as explants to initiate secondary somatic embryogenesis cultures; (i) & (iii) cotyledon explants on hormone-free medium at 28 days ACI, from cultures initiated on SCG medium containing 2, 4D and modified SCG without 2, 4D, respectively; (iv) & (v) cotyledon explants on ED at 46 days ACI (same treatments as above); (vi) & (vii) cotyledon explants on ED at 70 days ACI (same treatments as above); (Bars = 2 mm). **B**. Time course expression pattern of *TcLEC2* during cacao secondary somatic embryogenesis from cultures initiated on SCG medium containing 2,4D and modified SCG without 2, 4D. Expression of *TcLEC2* was normalized relative to that of *TcACP1* (n = 3 or 4, mean ± SE). **C**. Expression levels of *TcLEC2* at different time points in embryogenic and non-embryogenic calli. Expression of *TcLEC2* was normalized relative to that of *TcACP1*. Bars represent mean ± SE (n = 3 or 4). Significance was established by t-test (*represents for p-value < 0.05).

Based on the above results, we reasoned that TcLEC2 might also be a key regulator of somatic embryogenesis. To explore this, *TcLEC2* was measured in tissues grown with or without the SE inducing hormone 2,4-D (Figure 4). Staminodes from the highly embryogenic cacao genotype *PSU-SCA6* were used to produce primary somatic embryos (Figure 4A, panel i) following our previously published protocol [5]. Cotyledon explants (Figure 4A, panel i, red box) were excised and placed on secondary embryogenesis induction media with (SCG) or without (SCG-2,4D) auxin 2,4-D (required for SE induction). After two weeks on these media, the tissues were transferred biweekly to hormone free embryo development media (ED). The explants cultured on SCG media started to produce calli two-weeks after culture initiation (ACI) (panel ii) and secondary somatic embryos were visible after four additional weeks (panel iv and vi). However, on SCG-2,4D, explants expanded and gradually turned green during the first six weeks, then stopped developing and turned brown. Neither calli nor embryos were produced from the explants on SCG-2,4D medium (panel iii, v and vii).

*TcLEC2* expression levels were measured in tissues cultured on both SCG and SCG-2,4D media throughout the culture period (Figure 4B). *TcLEC2* expression was detectable in primary somatic embryo cotyledons at time 0, then decreased significantly one day after explants were placed on either SCG or SCG-2,4D media (Figure 4B). *TcLEC2* expression remained low in both treatments for the following two weeks, indicating that *TcLEC2* was not rapidly responsive to exogenous auxin treatment during the induction period. However, between day 32 and 36 ACI, *TcLEC2* expression levels were slightly increased and variable in both treatments. Notably, at 46 days ACI the development of embryos was first observed on SCG media arising from calli (embryogenic calli). RNA was extracted from the embryogenic calli (without visible embryos) and a large increase in *TcLEC2* gene expression was observed by qRT-PCR. On SCG-2,4D media, embryos were not observed and *TcLEC2* expression was not detectable.

A common occurrence in tissue culture is dedifferentiation of different types of calli that vary in their totipotency to regenerate somatic embryos [45,46]. With cacao tissue cultures, we and our collaborators have frequently observed two types of calli, those that produce abundant embryos (embryogenic calli) and those that produce few if any embryos (non-embryogenic calli) (unpublished observations). To investigate the relationship between TcLEC2 activity and embryogenic potential of the calli, *TcLEC2* gene expression was compared in embryogenic and non-embryogenic calli growing from explants cultured on SCG media. The observed average levels of *TcLEC2* expression were 20-fold higher in the embryogenic calli compared to the non-embryogenic calli of the same age (Figure 4C), suggesting a tight association between *TcLEC2* expression and embryogenic competency. Given the role of AtLEC2 in controlling embryo development in Arabidopsis, we hypothesized that TcLEC2 may play a similar role in the control of cacao somatic embryo development.

### Overexpression of *TcLEC2* significantly increased efficiency of somatic embryogenesis and regeneration of transgenic embryos

The current methods for *Agrobacterium*-mediated transformation of cacao genotype results in reproducible but very low rates of transgenic embryo recovery [47]. We speculate that this is a result of very low co-incidence of stable T-DNA integration into the cacao genome and the same cells entering the embryogenic pathway. We hypothesized that overexpression of *TcLEC2* might enhance the rate of somatic embryogenesis and thus improve the recovery of transgenic SEs through increased co-incidence with T-DNA integration events.

To test this, we performed *Agrobacterium*-mediated transformation experiments on cotyledon explants excised from primary embryos for co-cultivation with *Agrobacterium* containing the control vector (pGH00.0126) or the E12Ω::TcLEC2 vector (pGZ12.0108). Two weeks after co-cultivation, the initial transient expression levels of GFP in tissues transformed with the control vector were always higher than E12Ω::TcLEC2 (Additional file 5). This may be due to the larger size of the E12Ω::TcLEC2 containing plasmid relative to the control vector, the inclusion of a repeated promoter element, or the addition of a third highly expressed transgene. We have observed this phenomenon with other unrelated plasmids containing transgenes (unpublished data).

**Figure 5:**
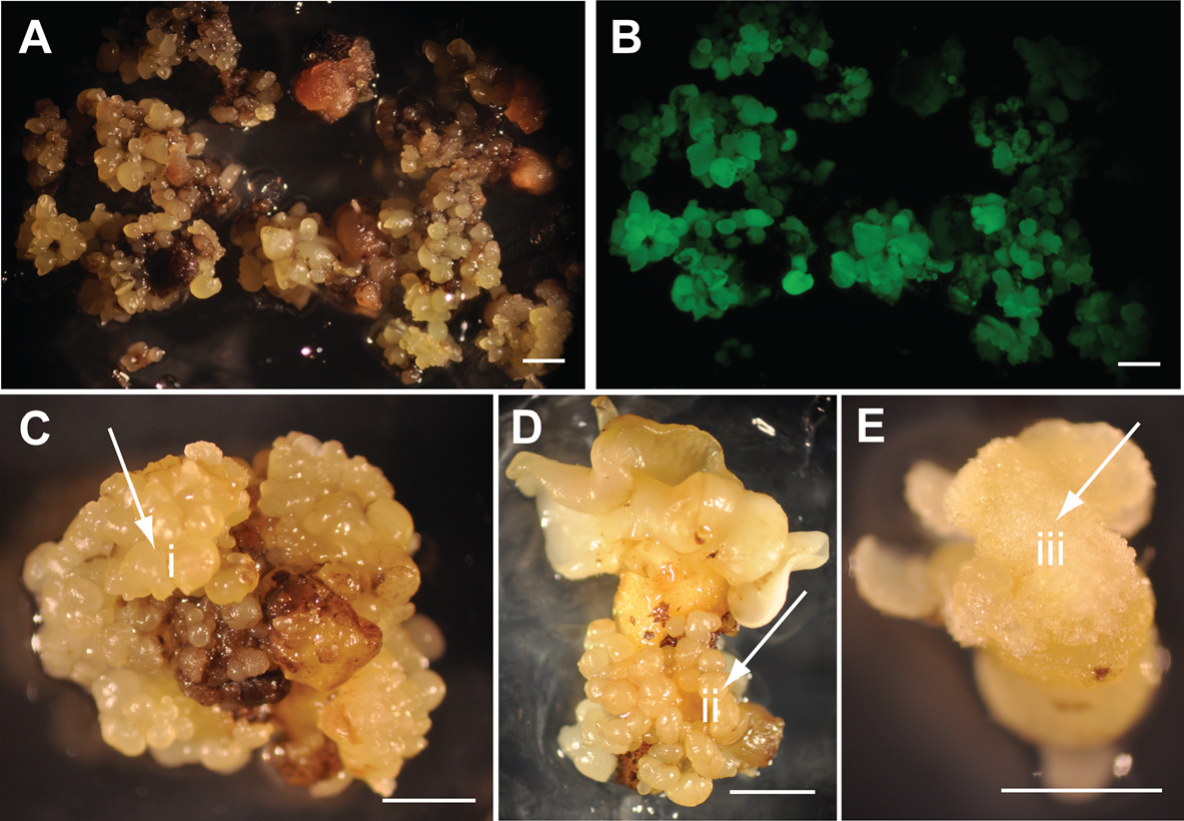
**Effect of stable overexpression of *TcLEC2 *in cacao secondary somatic embryos. A** &**B**. Secondary embryogenic explants transformed with *Agrobacterium*, regenerating stable transgenic E12Ω::TcLEC2 embryos were photographed under white and with GFP imaging optics, respectively. **C**, **D** &**E**. Transgenic somatic embryos expressing E12Ω::TcLEC2*.* (i) embryo-like structure formed on top of cotyledon (ii) embryo-like structure formed along embryo axis (iii) callus-like structure formed on top of cotyledon.

During the subsequent weeks of culture on embryogenesis media, large numbers of non-transgenic embryos (GFP negative) were observed in all three independent transformation trials regardless of the presence of the *TcLEC2* transgene (Additional file 6). There was no consistently significant difference observed between the transformations of control vector and E12Ω::TcLEC2 in terms of the cumulative non-transgenic embryo production (Additional file 6). To identify stably transformed embryos, GFP fluorescence was observed by stereomicroscopy as a visualization marker. With the control vector lacked the *TcLEC2* transgene, no GFP expressing embryos were observed on over 176 cotyledon explants cultured in three separate experiments. Surprisingly, the transformation with E12Ω::TcLEC2, containing the*TcLEC2* transgene, resulted in the recovery of over 300 stable transgenic embryos distributed over the entire surface of the cotyledon explant (Figure 5A-B). This result was dramatically higher than the stable transformation results we have observed over many years, with several different transgenes, where the prior record for a single transformation (about 200 cotyledon explants) was 8 GFP positive embryos [47-49]. Thus, although TcLEC2 did not impact the initial levels of transient transformation (Additional file 5) or embryogenesis frequency of non-transgenic embryos (Additional file 6), it greatly increased the frequency of transgenic embryo production, which confirmed our hypothesis.

Although a large number of transgenic *TcLEC2* embryos were obtained, most of them exhibited prominent developmental and morphological abnormalities (Figure 5C, D, and E), and most ceased development at the globular or heart stage and the initiations of cotyledons were significantly compromised. The few embryos that did develop to cotyledon stage formed callus on top of the cotyledons (Figure 5E) and new embryos were occasionally initiated along the embryo axis (Figure 5C and D). The attempts to recover plants from any of these embryos were unsuccessful.

**Figure 6:**
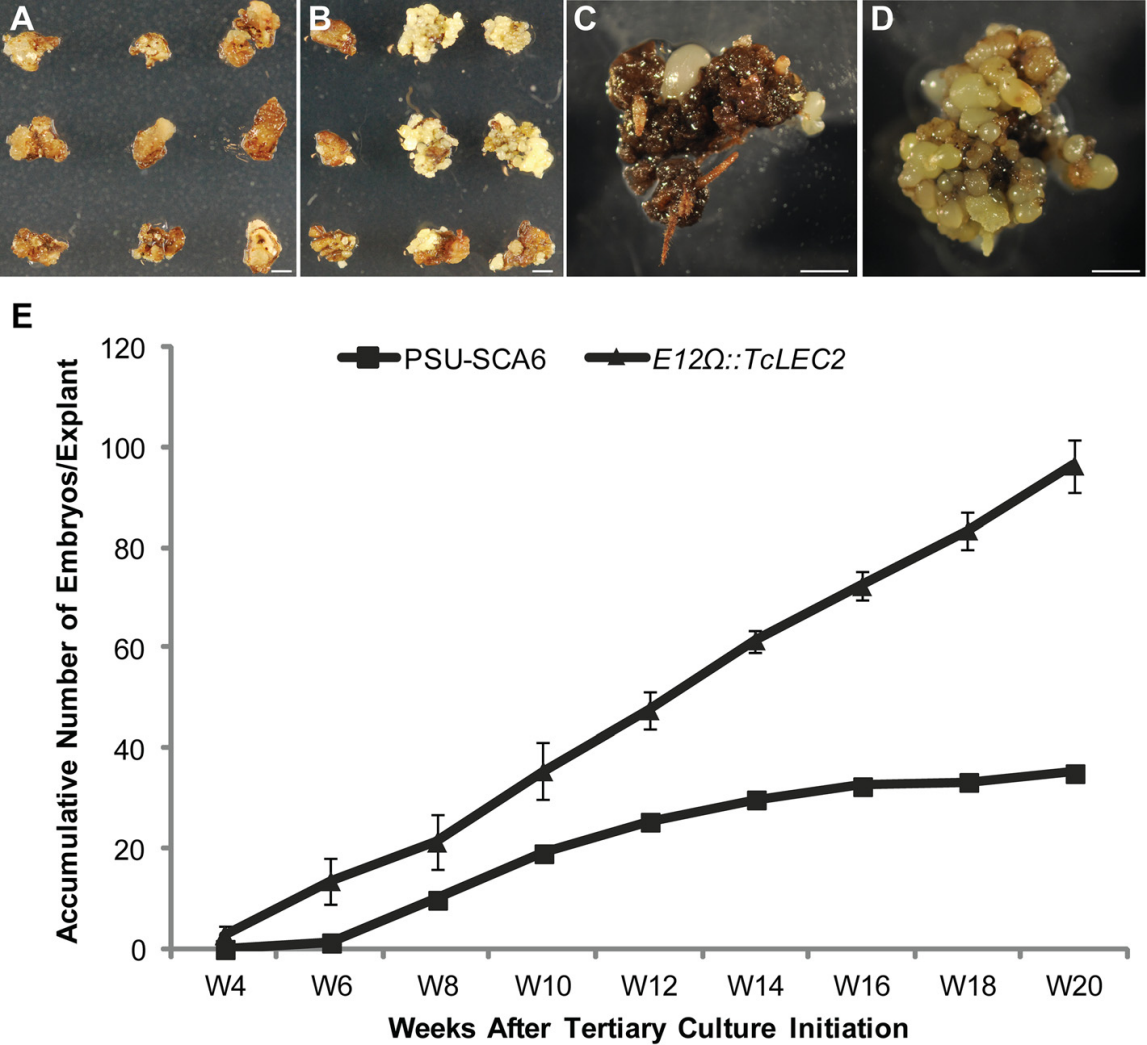
**Overexpression of *TcLEC2 *increases tertiary somatic embryogenesis efficiency. A**. Tertiary *PSU-SCA6* culture on hormone free medium at 4 weeks ACI. **B**. Tertiary stable transgenic E12Ω::TcLEC2 culture on hormone free medium at 4 weeks ACI. **C**. Tertiary *PSU-SCA6* culture on hormone free medium 20 weeks after culture initiation. **D**. Tertiary stable transgenic E12Ω::TcLEC2 culture on hormone free medium at 20 weeks ACI. **E**. Average number of tertiary embryos produced per explant from *PSU-SCA6* and stable transgenic E12Ω::TcLEC2 explants (n = 4, mean ± SE) (Bars = 2 mm).

To test the effect of stable overexpression of *TcLEC2* transgene on iterative somatic embryogenesis, cotyledons from fully developed mature transgenic E12Ω::TcLEC2 embryos were excised and cultured for tertiary embryo production as previously described [5]. Cotyledon explants from non-transformed *PSU-SCA6* SEs were cultured as controls. Remarkably, cotyledon explants from transgenic E12Ω::TcLEC2 lines started to produce tertiary embryos as early as four weeks ACI (Figure 6B), compared to six weeks for *PSU-SCA6* lines (Figure 6A). Additionally, while the majority of tertiary embryo production from *PSU-SCA6* lines was completed by 14 weeks ACI, after which very few SEs were produced (Figure 6C), explants from transgenic E12Ω::TcLEC2 lines continued to produce large numbers of embryos until twenty weeks ACI, when the experiment was terminated (Figure 6D). In total, within the twenty week period, transgenic E12Ω::TcLEC2 lines produced about 2.5 times more tertiary embryos per explant (p-value < 0.001) compared to *PSU-SCA6* lines (Figure 6E).

### Overexpression of *TcLEC2* altered the expression of genes involved in fatty acid biosynthesis

In addition to its role in initiation of embryogenesis, it has been well documented in Arabidopsis that AtLEC2 also regulates de novo fatty acid biosynthesis during embryo development. Evidence includes, but is not limited to, (a) transgenic 35S::AtLEC2 ovules exhibited a mature seed-like fatty acid profile [24]; (b) ectopic overexpression of *AtLEC2* in leaves resulted in accumulation of seed specific lipids and very long chain fatty acids [16]; (c) *AtLEC2* directly regulates expression of *AtWRI1,* which is known to play a role in regulation of fatty acid metabolism in developing embryos [17]. Since fatty acids, in the form of triacylglycerols (TAGs), are major storage components of mature cacao seeds, we examined the role of TcLEC2 in control of fatty acid biosynthesis in cacao immature zygotic embryos (IZEs).

**Figure 7:**
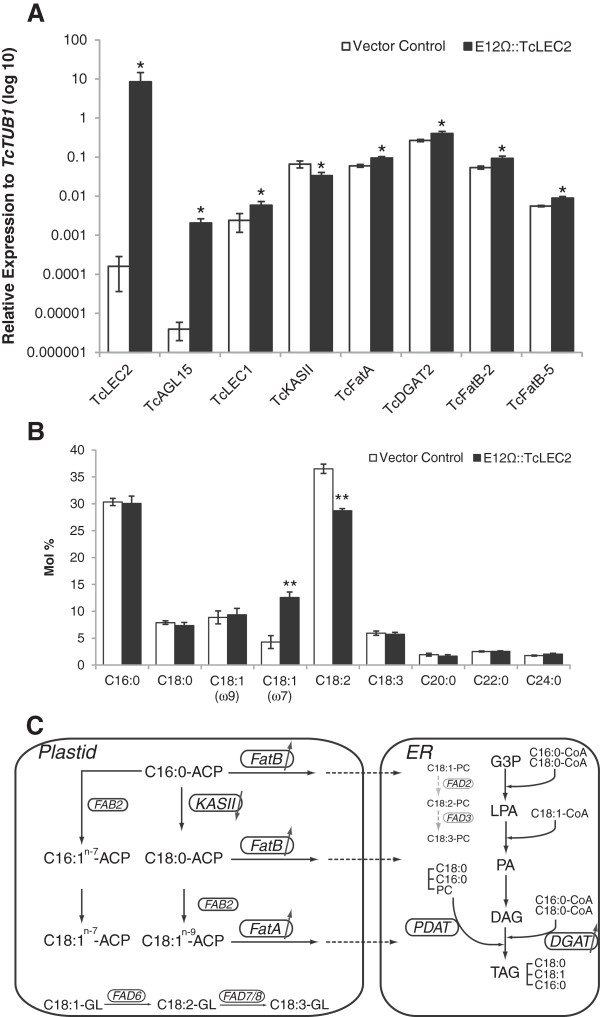
**Transient overexpression of E12Ω::TcLEC2 in IZE altered fatty acid compositions and gene expression. A**. Changes of the expression levels of responsive enzymes on the fatty acid biosynthesis pathway; expression levels of genes were normalized relative to that of *TcTUB1*; (n = 3, mean ± SE) *represents for p-value < 0.05 by t-test. **B**. Molar percentages of fatty acid compositions in cacao immature zygotic embryos transiently overexpressing vector control and E12Ω::TcLEC2, respectively; (n = 3, mean ± SE). **represents for p-value < 0.001 by t-test. **C**. Diagram of proposed model to explain the relationship between gene expression levels and altered fatty acid compositions. Enzymes are marked in circle. Enzymes that were regulated by the activity of TcLEC2 are in black, otherwise, in grey. Abbreviation: ER, endoplasmic reticulum; ACP, acyl carrier protein; CoA, Coenzyme A; FAB2, fatty acid desaturase; Fat, fatty acyl-ACP thioesterase; KAS, 3-ketoacyl-ACP synthase; FAD2, oleoyl desaturase; FAD3, linoleoyl desaturase; FAD6, oleoyl desaturase on membrane glycerolipid; FAD7/8, linoleoyl desaturase on membrane glycerolipids; PC, phosphatidylcholine; G3P, glycerol-3-phosphate; LPA, lysophosphatidate; PA, phosphatidate; DAG, diacylglycerol; TAG, triacylglycerol; PDAT, phospholipid:diacylglycerol acyltransferase; DGAT, 1,2-sn-diacylglcyerol transferase.

E12Ω::TcLEC2 was transiently overexpressed in IZEs (12 weeks old) in parallel with the control vector (pGZ00.0126). High transient expression was confirmed by fluorescence microscopy to detect EGFP on approximately 90% of explants surfaces (Additional file 7). Overexpression levels of *TcLEC2* in transformed IZEs were further confirmed by qRT-PCR and compared to the basal levels of *TcLEC2* in control vector transformed IZEs (Figure 7). Consistent with the observations in attached leaf transient assay (Figure 3), the overexpression of *TcLEC2* resulted in elevated transcript of *TcAGL15* and *TcLEC1* in the IZE tissues (Figure 7A). Unlike in transiently transformed leaf tissue, induced expression of *TcWRI1* was not detected in the transformed IZEs under our qRT-PCR condition (40 cycles) (Additional file 8).

To obtain further insights into TcLEC2 regulatory functions during embryo development, we identified the most likely orthologs of genes for key enzymes controlling the fatty acid biosynthesis and production of TAGs in the cocoa genome by homology to Arabidopsis gene sequences (Figure 7C and Additional file 9) and compared their expression levels in IZEs tissues overexpressing E12Ω::TcLEC2 and control vector (Figure 7A). mRNA levels of *TcKASII* (*Tc09g006480*), a condensing enzyme β-ketoacyl-[acyl-carrier-protein] synthase II responsible for the elongation of C16 to C18 [50], was two-fold lower (p-value < 0.05) in the *TcLEC2* transformed tissue compared to the controls (Figure 7A). In addition, the predicted ortholog of *TcFatA* (*Tc01g022130*) and two isoforms of *TcFatB* (*Tc01g022130* and *Tc03g015170*), two types of acyl-[acyl-carrier-protein] thioesterases that specifically export C18:1 (FatA) and other saturated fatty acid moieties (FatB) from plastid into cytosol [51], were significantly up-regulated by more than 1.5 fold (p-value < 0.05). Interestingly, the predicted diacylglycerol acyltransferase 2 (*TcDGAT2, Tc01g000140*), a key enzyme that catalyzed the last step of TAG assembly through an acyl-CoA dependent pathway [52], was significantly up-regulated by 1.5 fold (p-value < 0.05). No significant differences in the expression levels of two isoforms of fatty acid desaturase 2 (*FAB2, Tc04g017510 and Tc08g012550*) were observed (Additional file 8).

To determine if these changes in gene expression resulted in altered metabolite profiles, fatty acid composition was measured by gas chromatograph/mass spectrometry (GC/MS) in IZEs tissues transformed with both E12Ω::TcLEC2 and control vector. Overexpression of *TcLEC2* resulted in a significant increase of the level of cis-vaccenic acid C18:1^n-7^ (p-value < 0.001), an isoform of oleic acid (OA), and significantly decreased the level (p-value < 0.001) of linoleic acid (LA, C18:2^n-6^) compared to tissues transformed with vector control (Figure 7B).

## Discussion

### TcLEC2 is involved in cacao somatic embryogenesis

Somatic embryogenesis has long been considered a superior propagation system for many crops [53-55] because of its inherent high multiplication rate and potential for year round, uniform disease free plant production. Although theoretically, every somatic plant cell has the capacity to dedifferentiate and redifferentiate into a whole plant (totipotency), the competencies of plant cells to enter the somatic embryogenesis developmental pathway varies dramatically between different tissues, developmental stages, and species. Accumulated evidence has revealed that the activity of AtLEC2 is highly associated with embryogenic competency and involves interactions with several other regulatory factors. Our results are consistent with a role of cacao TcLEC2 in the regulation of somatic embryogenesis similar to AtLEC2 in Arabidopsis. Supporting evidence includes; (1) ectopic overexpression of *TcLEC2* in cacao stage C leaves was able to induce the expression of seed transcription factor genes, such as *TcAGL15*, *TcABI3* and *TcLEC1;* (2) the induced expression level of *TcLEC2* was associated with embryogenic capacity in explants; (3) constitutive overexpression of *TcLEC2* in secondary somatic embryo tissue leads to earlier and increased regeneration of tertiary embryos compared to *PSU-SCA6* controls. Collectively, our evidence supports the conclusion that TcLEC2 is a functional ortholog of AtLEC2 and that it is involved in similar genetic regulatory networks during cacao somatic embryogenesis.

Transient overexpression of *TcLEC2* in cotyledon explants by itself was not sufficient to increase embryogenesis efficiency of non-transgenic somatic embryos (Additional file 6). This suggests that there are other factor (s) that are required for cell dedifferentiation and redifferentiation, which are not present during the period of time examined in our embryogenesis culture system. However, the constitutive overexpression of *TcLEC2* in stably transformed cells resulted in greatly enhanced somatic embryogenesis as early as four weeks compared to six to seven without *TcLEC2* overexpression (Figures 5B, 6B and E), implying that the enhanced activity of TcLEC2 is sufficient to promote the efficiency of somatic embryogenesis in cacao.

The very high degree of genotype variation in embryogenic capacity for SE in cacao limits its’ practical application for large scale propagation [5]. Therefore, *TcLEC2* could be a useful molecular marker for screening cacao genotypes for high embryogenic capacities. Additionally, the levels of *TcLEC2* expression in callus and other tissues *in vitro* could be used for evaluating the effect of different media and other variables for further optimization of the SE protocols. Potentially, we could explore the possibility to promote somatic embryogenesis in cacao leaves or other tissues by ectopically expressing *TcLEC2*.

### TcLEC2 regulates fatty acid biosynthesis during cacao seed maturation

Fatty acid composition and lipid profiles of cacao seeds are important quality traits for chocolate industry. Therefore, there is great interest in identification of the genetic networks regulating its biosynthesis. LEC2, and its partners LEC1, ABI3 and FUS3 are known to be critical regulators of fatty acid and lipid biosynthesis in Arabidopsis and other species, and thus impact many aspects of seed development. Moreover, of particular relevance to applications of this knowledge, the level of WRI1, a downstream target of LEC1, LEC2 and FUS3, was highly correlated with seed oil content in different *B. napus* genotypes [28]. Our observations that *TcLEC2* overexpression resulted in increased expression of *TcLEC1* and *TcWRI1* (Figure 3) in attached cacao leaves promoted us to speculated that this might result in changes in fatty acid composition and TAG assembly. Indeed, transient overexpression of *TcLEC2* in zygotic embryos resulted in increased C18:1^n-7^ and decreased C18:2^n-6^ levels (Figure 7B), similar to changes occurring during cacao seed maturation when profiles change from mainly polyunsaturated fatty acids (C18:2^n-6^ and C18:3^n-3^) to almost exclusively saturated (C16:0 and C18:0) and monounsaturated fatty acid (C18:1^n-9^) [36]. However, given the fact that the expression of *TcWRI1* was not induced by overexpression of *TcLEC2* in immature zygotic embryos, it suggests that WRI1 is not required to mediated impacts of TcLEC2 on fatty acid biosynthesis, and that the regulatory network between TcLEC2 and other transcription factors on fatty acid biosynthesis is not the same in cacao as they are in Arabidopsis and *B. napus*. The overexpression of *TcLEC2* also resulted in changes in gene expression for some of the major structural genes for fatty acid biosynthesis, and this could provide an explanation for the fatty acid composition shifts we observed. C18:1^n-7^ is synthesized from C16:0 via the production of C16:1^n-9^ by FAB2 and further elongation to C18:1^n-7 ^[56]. The decreased expression level of *TcKASII* may increase the substrate availability of C16:0, which could serve as a substrate for TcFAB2 for the production of C16:1^n-9^ and further leading to C18:1^n-7^ accumulation (Figure 7C). The increased levels of *TcFatA* and two isoforms of *TcFatB* (all significantly up-regulated by more than 1.5 fold) could contribute to increased production and accumulation of saturated fatty acid (C16:0 and C18:0) and monounsaturated fatty acid (C18:1^n-9^) during cacao seed maturation (Figure 7C).

Interestingly, the expression of *TcDGAT2* was also significantly increased by overexpression of *TcLEC2*, but the expression level of *TcDGAT1.1* was not affected (Additional file 8). The activities of DGAT genes were highly correlated with the oil content and compositions in oilseeds [57] and three known types of DGAT genes (DGAT1, DGAT2 and DGAT3) are different in terms of substrate specificities and subcellular localizations [58]. According to an unpublished study, the expression of *TcDGAT2* in yeast has led to accumulation of more C18:0 in TAG fraction compared to the expression of *TcDGAT1 *[59]. Considering the fact that the majority of TAGs in cacao mature seeds consist of unsaturated fatty acid (C18:1) exclusively on sn-2 and saturated fatty acids (C16:0 and C18:0) on sn-1 and 3 (Figure 7C), it is plausible to speculate, that the activity of TcDGAT2 is more significant to catalyze the final acylation on sn-3 of TAG assembly compared to TcDGAT1. This argument was further supported by our result indicating that the expression level of *TcDGAT2* was approximately five times higher than *TcDGAT1* in cacao immature seeds (Figure 7A and Additional file 8). Collectively, our data indicates that TcLEC2 could be involved in regulation of lipid biosynthesis during cacao seed maturation through control of *TcDGAT2* gene expression. However, whether TcLEC2 is able to directly trans-activate *TcDGAT2* or its action is mediated through other transcription factors, remains unknown. Further research on the regulatory mechanism controlling fatty acid biosynthesis and TAG assembly in cacao will contribute to identification of the key enzymes in the pathway and aid the screening process for elite cacao varieties to meet industrial demands.

## Conclusion

The isolation and functional characterization of *LEC2* ortholog from cacao genome reveal crucial roles of TcLEC2 in regulating both zygotic and somatic embryogenesis. The exclusive expression pattern in seed and the identification of its regulatory targets, such as *AGL15* and *WRI1*, strongly indicate the functional similarities between AtLEC2 and TcLEC2. However, the impacts of TcLEC2 on fatty acid biosynthesis in cacao also suggest that TcLEC2 is able to direct or indirectly interact with many key enzymes on the pathway, which has not been well characterized yet in Arabidopsis. Furthermore, the correlation between the activity of TcLEC2 and embryogenic potential during cacao somatic embryogenesis provides us a great opportunity to better understand and improve our current inefficient and variable propagation system of cacao.

## Methods

### Phylogenetic analysis and sequence alignment

B3 domain containing genes in *Theobroma cacao* were identified by blastp using AtLEC2 (At1g28300) as queries (E-value cut off 1e^-5^). Multiple protein sequence alignment was performed by MUSCLE [60]. The phylogenetic tree was constructed by MEGA4.1 using neighbor-joining algorithm with Poisson correction model and the option of pairwise deletion [61]. Bootstrap values represent 1000 replicates. Full-length Arabidopsis *AtLEC2, AtABI3,* and *AtFUS3,* protein sequences were used to search the Cocoa Genome Database (http://cocoagendb.cirad.fr/) by tblastn [33] to obtain the full-length *TcLEC2, TcABI3* and *TcFUS3* nucleotide sequences, respectively. The functional B3 domains were predicted using InterPro program (http://www.ebi.ac.uk/interpro/) on EMBL-EBI website. B3 domain containing proteins from five subfamilies in *Arabidopsis* were identified and selected according to [31].

### RNA extraction, *TcLEC2* cloning and expression vector construction

Plant tissues collected from SCA6 genotype of cacao were first ground in liquid nitrogen. Total RNA was extracted using Plant RNA Purification Reagent (Life Technologies, Cat. 12322–012, following manufactures protocol). The concentration of RNA was measured using a Nanodrop 2000c (Thermo Scientific). RNA was further treated with RQ1 RNase-free DNase (Promega, Cat. M6101) to remove potential genomic DNA contamination (following the manufacturer’s protocol). 250 ng of treated RNA was reverse-transcribed by M-MuLV Reverse Transcriptase (New England Biolabs) with oligo-(dT)15 primers. The full length *TcLEC2* was amplified from *SCA6* mature seed cotyledon cDNA with the primer pair (*TcLEC2*-*5′*-SpeI: GCACTAGTATGGAAAACTCTTACACACC and *TcLEC2-3′*-HpaI: GCGTTAACTCAAAGTGAAAAATTGTAGTGATTGAC) and cloned into pGH00.0126 [47] driven by the E12-Ω promoter resulting in plasmid pGZ12.0108 (Additional file 7). The recombinant binary plasmid was introduced into *A. tumefaciens* strain *AGL1 *[62] by electroporation.

### *TcLEC2* expression analysis by qRT-PCR

RNA samples were extracted and reverse-transcribed into cDNA as described above. The primers to detect *TcLEC2* transcripts were designed based on the coding sequence of *TcLEC2 (Tc06g015590 *[32]*)* (*TcLEC2-Realtime-5′*: TGACCAGCTCTGGTGCTGACAATA; *TcLEC2-Realtime-3′*: TGATGTTGGGTCCCTTGGGAGAAT). qRT-PCR was performed in a 10 μl mixture containing 4 μl diluted-cDNA (1:50), 5 μl SYBR Green PCR Master Mix (Takara), 0.2 μl Rox, and 0.4 μl each 5 μM primers. Each reaction was performed in duplicates in Roche Applied Biosystem StepOne Plus Realtime PCR System under the following program: 15 min at 94°C, 40 cycle of 15 s at 94°C, 20s at 60°C, and 40 s at 72°C. The specificity of the primer pair was examined by PCR visualized on a 2% agarose Gel and dissociation curve. An acyl carrier protein (*Tc01g039970*, *TcACP1 *[32] *TcACP1-5′*: GGAAAGCAAGGGTGTCTCGTTGAA and *TcACP1-3′*: GCGAGTTGAAATCTGCTGTTGTTTGG), and a tubulin gene in cacao (*Tc06g000360, TcTUB1 *[32] *TcTUB1-5′*: GGAGGAGTCTCTATAAGCTTGCAGTTGG and *TcTUB1-3′*: ACATAAGCATAGCCAGCTAGAGCCAG) were used as the reference genes.

### Cacao attached leaf and immature zygotic embryo transient gene expression assay

*A. tumefaciens* strain *AGL1* carrying either control vector (pGH00.0126, GenBank Accession: KF018690, EGFP only) or E12Ω::TcLEC2 (pGZ12.0108, GenBank Accession: KF963132, Additional file 4) were inoculated in 100 ml 523 medium with 50 μg/ml kanamycin and grown with shaking (200 rpm, 25°C) overnight to optical density (O.D.) of 1.0 at 420 nm. *AGL1* was pelleted at 1500xg for 17 min at room temperature and resuspended in induction media [63] to to O.D. of 1.0 at 420 nm. *AGL1* was induced for 3 h at 100 rpm at 25°C and Silwet added to a final concentration of 0.02%. For the attached leaf transient transformation assay we used fully expanded, young leaves (developmental stage C as defined in [64]) from genotype *SCA6* grown in a greenhouse. The petioles of the leaves were wrapped with parafilm and set in the groove of a modified vacuum desiccator to create a seal and to avoid damage to leaves. The leaves were soaked in AGL1 induction media in the desiccator and were vacuum infiltrated at -22 psi for 2 min using a vacuum pump (GAST Model No. 0523-V4F-G582DX). Vacuum infiltration was performed three times to increase transformation efficiency. The transformed cacao leaves remained on the plant for three days after infiltration then collected and evaluated by fluorescence microscopy. The regions with high GFP expression (>80% coverage) were selected and subjected to further analysis. For immature zygotic embryo transient transformation assay, developing fruit (open pollinated *Sca6*, four months after pollination) from the USDA germplasm collection in Puerto Rico. Zygotic embryo cotyledons were collected and suspended in the ED media [5] before transformation. Zygotic cotyledons were soaked in AGL1 induction media and transformation was performed as described above for leaf transient expression assays. The transformed tissues were analyzed five days after infiltration.

### Cacao stable transformation of primary somatic embryos

Primary somatic embryogenesis was performed as previously described [8]. Glossy cotyledons from healthy and mature primary embryos were cut into 4 mm X 4 mm square pieces, and infected using *A. tumefaciens* strain AGL1 carrying the T-DNA binary vectors as previously described [47] with minor modifications: (1) the AGL1 was pelleted and resuspended in induction media [63] to reach the O.D. of 1.0 at 420 nm instead of 0.5; (2) after transformation, the infected cotyledons were co-cultivated with *A. tumefaciens* strain AGL1 on the filter paper for 72 h at 25°C in the dark instead of 48 h. The transformed explants were cultivated and transgenic secondary somatic embryos were identified by screening for GFP fluorescence as previously described [47].

### Fatty acid profiling by GC/MS

Fresh plant tissues were ground in liquid nitrogen and fatty acid methyl esters (FAME) were prepared using approximately 30 mg of tissue extracted in 1 ml buffer containing MeOH/fuming HCl/Dichloromethane (10:1:1, v/v) while incubated without shaking at 80°C for 2 h. Fatty acid methyl esters were re-extracted in 1 ml buffer H2O/Hexane/Dichloromethane (5:4:1, v/v) with vortexing for 1 min. The hexane (upper phase) was separated by centrifugation at 1500xg for 5 min, transferred to Agilent glass GC vials and evaporated to dryness under a vacuum. The FAMEs were then dissolved in 500 μl hexane for GC/MS analysis. Pentadecanoic acid (C15:0) (Sigma, Cat. P6125) was used as the internal standard added prior to the extraction and methyl nonadecanoate (C19:0-methyl ester) (Sigma, Cat. N5377) was used as the spike control, added into the sample prior to the GC injection. Fatty acid derivatives were analyzed on an Agilent 6890 Gas Chromatograph equipped with FAME Mix Omegawax 250 Capillary GC column (Sigma, Cat. 24136). A Waters GCT Classic mass spectrometry was directly connected to the GC operation. EI of 70 eV was applied. Peak height areas were used to quantify the abundance of each fatty acid species, and the mass spectra were interpreted by comparing with the NIST/EPA/NIH Mass Spectra Library [65].

### Accession numbers

Sequence data from this article can be found in either The Arabidopsis Information Resource (TAIR) or CocoaGenDB (http://cocoagendb.cirad.fr/gbrowse/cgi-bin/gbrowse/theobroma/) under the following accession numbers in Table 2.

**Table 2:** Accession numbers of tested genes in our study

**Gene**	**Database**	**Accession number**	**Gene**	**Database**	**Accession number**
AtLEC1	TAIR	AT1G21970	TcFatB5	CocoaGenDB	Tc03g015170
AtLEC2	TAIR	AT1G28300	TcKASII	CocoaGenDB	Tc09g006480
AtABI3	TAIR	AT3G24650	TcFAD2.1	CocoaGenDB	Tc05g018800
AtFUS3	TAIR	AT3G26790	TcFAD2.2	CocoaGenDB	Tc05g018800
AtAGL15	TAIR	AT5G13790	TcFAD3	CocoaGenDB	Tc09g029750
AtWRI1	TAIR	AT3G54320	TcFAD6	CocoaGenDB	Tc09g029750
TcLEC1	CocoaGenDB	Tc07g001180	TcFAD7/8	CocoaGenDB	Tc05g002310
TcLEC1-like	CocoaGenDB	Tc06g020950	TcDGAT1.1	CocoaGenDB	Tc09g007600
TcLEC2	CocoaGenDB	Tc06g015590	TcDGAT1.2	CocoaGenDB	Tc01g035170
TcABI3	CocoaGenDB	Tc01g024700	TcDGAT2	CocoaGenDB	Tc01g000140
TcFUS3	CocoaGenDB	Tc04g004970	TcPDAT1	CocoaGenDB	Tc09g029110
TcAGL15	CocoaGenDB	Tc01g040120	TcTUB1	CocoaGenDB	Tc06g000360
TcWRI1	CocoaGenDB	Tc10g012790	TcACP1	CocoaGenDB	Tc01g039970
TcOLE1	CocoaGenDB	Tc04g001560	TcVicilin	CocoaGenDB	Tc04g024090
TcOLE2	CocoaGenDB	Tc09g004410	TcBBM	CocoaGenDB	Tc05g019690
TcFAB2.2	CocoaGenDB	Tc04g017510	TcPKL	CocoaGenDB	Tc09g001610
TcFAB2.7	CocoaGenDB	Tc08g012550	TcWUS	CocoaGenDB	Tc01g001780
TcFatA	CocoaGenDB	Tc01g022130	TcYUC2	CocoaGenDB	Tc09g009820
TcFatB1	CocoaGenDB	Tc09g010360	TcYUC4	CocoaGenDB	Tc09g013260
TcFatB2	CocoaGenDB	Tc01g022130			

## Abbreviations

SCA6: Scavana 6; LEC: Leafy cotyledon; ABI3: ABA INSENSITIVE 3; FUS3: FUSCA3; SE: Somatic embryogenesis; ZE: Zygotic embryogenesis; WRI1: WRINKLED 1; AGL15: AGAMOUS-like 15; OLE: OLEOSIN; YUC: YUCCA; ARF: Auxin response factor; HSI: High level expression of sugar inducible; RAV: Related to ABI3/VP1; REM: Reproductive meristem; TUB1: Tubulin; ACP1: Acyl carrier protein; 2, 4D: 2, 4-Dichlorophenoxyacetic acid; SCG: Secondary embryogenesis induction media; ACI: After culture initiation; IZE: Immature zygotic embryo; TAG: Triacylglycerol; GC/MS: Gas chromatograph/mass spectrometry; OA: Oleic acid; LA: Linoleic acid; ER: Endoplasmic reticulum; CoA: Coenzyme A; FAB2: Fatty acid desaturase; Fat: Fatty acyl-ACP thioesterase; KAS: 3-ketoacyl-ACP synthase; FAD2: Oleoyl desaturase; FAD3: Linoleoyl desaturase; FAD6: Oleoyl desaturase on membrane glycerolipid; FAD7/8: Linoleoyl desaturase on membrane glycerolipids; PC: Phosphatidylcholine; G3P: Glycerol-3-phosphate; LPA: Lysophosphatidate; PA: Phosphatidate; DAG: Diacylglycerol; TAG: Triacylglycerol; PDAT: Phospholipid:diacylglycerol acyltransferase; DGAT: 1, 2-sn-diacylglcyerol transferase.

## Competing interests

The authors declare that they have no competing interests.

## Authors’ contributions

YZ performed most of the experiments, such as phylogenetic analysis, gene expression analysis, transient and stable transformation assays, FAME analysis, and drafted the manuscript. AC participated in the vector construction, gene expression analysis, somatic embryogenesis transformation, and review the manuscript. SNM involved in designing and directing the experiments, and revising the manuscript. MJG conceived the study, gave advice on experiments, drafted and finalized the manuscript. All authors read and approved the final manuscript.

## Supplementary Material

Additional file 1 Correspondent gene comparison from Criollo and Forastero genome database. Click here for file

Additional file 2 **Full-length amino acid alignment of TcLEC2, AtLEC2, AtFUS3, and AtABI3.** Residues in black boxes are identical in all four proteins; residues in dark grey boxes are identical in three of four proteins; residues in light grey boxes are identical in two of four proteins. Click here for file

Additional file 3 **Ectopic overexpression of control vector (pGH00.0126) and E12Ω::TcLEC2 in cacao attached leaf transient assay.** Fluorescent micrographs of GFP expression (visualization marker) in leaves were captured three days after transformation (Bars = 0.4mm). **A**. GFP fluorescence image of cacao stage C leaves transformed with control vector. **B**. GFP fluorescence image of cacao stage C leaves transformed with E12Ω::TcLEC2. Click here for file

Additional file 4 **Vector map of E12Ω::TcLEC2.** Location of the TcLEC2 and GFP transgenes are indicated as are the NPTII selectable marker genes, and the location of all plant promoter and terminator elements. The control vector plasmid (pGH00.0126, GenBank: KF018690.1) is identical but lacks the E12Ω-TcLEC2-35S Terminator transgene segment. Click here for file

Additional file 5 Relative transient GFP expression levels of TcLEC2 transformation in SE compared to PSUSCA6. Click here for file

Additional file 6 **Comparison of average of total number of non-transgenic embryo produced per cotyledonary explant.** Sixteen pieces of cotyeldonary explants were placed on each media plate. Three or four plates (taken as biological replicates) were used for transient transformation of control vector or E12Ω::TcLEC2 in each transformation trial (n=3 or 4, mean ± SE). **A**. Transformation trial 1 (n=3). **B**. Transformation trial 2 (n=4). **C**. Transformation trial 3 (n=4). Click here for file

Additional file 7 **Overexpression of control vector and E12Ω::TcLEC2 in cacao zygotic embryo transient assay.** Fluorescent micrographs of GFP expression (visualization marker) in leaves were captured five days after transformation (Bars = 2mm). **A** &**B**. IZE transformed with control vector with white light and GFP fluorescence imaging. **C** &**D**. IZE transformed with E12Ω::TcLEC2 with white light and GFP fluorescence imaging. Click here for file

Additional file 8 **Expression levels of genes that are not significantly affected by transient overexpression of *TcLEC2 *in cacao IZE compared to control vector (n=3, mean ± SE, significant levels were determined by t-test).** The gene encoding TcWRI1 was also measured but no expression was detected. Click here for file

Additional file 9 **List of fatty acid biosynthesis related genes in cacao.** The expression of these genes were compared in cacao IZE transiently overexpressing control vector and E12Ω::TcLEC2. Click here for file
