# Supplementary material for: The Theobroma cacao B3 domain transcription factor TcLEC2 plays a duel role in control of embryo development and maturation
Source: BMC Plant Biol. 2014 Apr 24;14:106. doi: 10.1186/1471-2229-14-106 (PMC4021495; doi:10.1186/1471-2229-14-106)
Supplement: Additional file 1 — Correspondent gene comparison from Criollo and Forastero genome database. [file 1471-2229-14-106-S1.pdf]

Additional file 1. Correspondent gene comparison from Criollo and Forastero genome database.

Criollo Genes	Forastero Genes	E-values
Tc06_g015590	Thecc1EG029838	0
Tc04_g004970	Thecc1EG017321	4.00E-159
Tc01_g024700	Thecc1EG003056	0
Tc09_g035210	Thecc1EG042290	0
Tc04_g016290	Thecc1EG019701	0
Tc06_g016770	Thecc1EG029976	0
Tc07_g007150	Thecc1EG031311	0
Tc00_g048490	Thecc1EG005014	0
Tc05_g010460	Thecc1EG022729	2.00E-105
Tc02_g034560	Thecc1EG011862	0
Tc03_g022470	Thecc1EG015590	0
Tc01_g015370	Thecc1EG040397	0
Tc04_g015240	Thecc1EG019508	0
