# Supplementary material for: The Theobroma cacao B3 domain transcription factor TcLEC2 plays a duel role in control of embryo development and maturation
Source: BMC Plant Biol. 2014 Apr 24;14:106. doi: 10.1186/1471-2229-14-106 (PMC4021495; doi:10.1186/1471-2229-14-106)
Supplement: Additional file 2 — Full-length amino acid alignment of TcLEC2, AtLEC2, AtFUS3, and AtABI3. Residues in black boxes are identical in all four proteins; residues in dark grey boxes are identical in three of four proteins; residues in light grey boxes are identical in two of four proteins. [file 1471-2229-14-106-S2.pdf]

Additional file 2. **Full-length amino acid alignment of TcLEC2, AtLEC2, AtFUS3, and AtABI3.** Residues in black boxes are identical in all four proteins; residues in dark grey boxes are identical in three of four proteins; residues in light grey boxes are identical in two of four proteins.

```
TcLEC2 : -----------------------------------------------------------------------------------------
AtLEC2 : -----------------------------------------------------------------------------------------
AtFUS3 : -----------------------------------------------------------------------------------------
AtABI3 : MKSLHVAANAGDLAEDCGILGGDADDTVLMDGIDEVGREIWLDDHGGDNNHVHGHQDDDLIVHHDPSIFYGDLPTLPDFPCMSSSSSSS

TcLEC2 : -----------------------------------------------------------------------------------------
AtLEC2 : -----------------------------------------------------------------------------------------
AtFUS3 : -----------------------------------------------------------------------------------------
AtABI3 : TSPAPVNAIVSSASSSSAASSSTSSAASWAILRSDGEDPTPNQNQYASGNCDDSSGALQSTASMEIPLDSSQGFGCGEGGGDCIDMMET

TcLEC2 : -----------------------------------------------------------------------------------------
AtLEC2 : -----------------------------------------------------------------------------------------
AtFUS3 : -----------------------------------------------------------------------------------------
AtABI3 : FGYMDLLDSNEFFDTSAIFSQDDDTQNPNLMDQTLERQEDQVVVPMMENNSGGDMQMMNSSLEQDDDLAAVFLEWLKNNKETVSAEDLR

TcLEC2 : ----------------------------------------------------MENSYTPFIPTTTTPTNTTLRQPDMGWSQNSEFSIYE
AtLEC2 : -----------------------------------------------------MDNFLPFPSSNANSV--------------QELSMDP
AtFUS3 : --------------------------------------------------MMVDENVETKASTLVASV---------------------
AtABI3 : KVKIKKATIESAARRLGGGKEAMKQLLKLILEWVQTNHLQRRRTTTTTTNLSYQQSFQQDPFQNPNPNNNNLIPPSDQTCFSPSTWVPP

TcLEC2 : PMNLKNESSTEFSTASNSQFHHPPQFVAESQSLQPPYRHEIEQAGHSL---------AYPMYPFFLGQNGIEYGVSSSIHANNGVQGSC
AtLEC2 : NNNRSHETTVETYDHHQAQPHH----FLE------PFSYEVEQMAAVMNPQPVYLSECYPQIP--VTQTGSEFGS--------------
AtFUS3 : ---------IHGFGSGSGHDHHGLSASVELL----------------------------------------------------------
AtABI3 : PPQQQAEVSDEGFGYMPAPNYPPQPEFLELLESPPSWPPEPQSGPMPHQQFPMPPTSQYNQFGDPTGFNGYNMNPYQYPYVPAGQ----

TcLEC2 : FMQTDGLSKEQERRIMDPYRTKVARIKRKLARQRSLSLQRNASSGA----------------------------------STQVDARRL
AtLEC2 : -LVGNPCLWQERGGFLDPRMTKMARINRKNAMMRSRNNSSPNSSPS----------------------------------ELVDSKRQL
AtFUS3 : -----GVNW---------KKRRMPRQRRSSSSF-----------------------------------------------NLLSFPPPM
AtABI3 : -MRDQRLLRLCSSATKEARKKRMARQRRFLSHHHRHNNNNNNNNNNQQNQTQIGETCAAVAPQLNPVATTATGGTWMYWPNVPAVPPQL

TcLEC2 : TSSGADNSTDVNNSNDTKRDLYKFCTPDNKR-------LRVLLRKELKNSDVGSLGRIVLPKREAEGNLPTLSDKEGIQVMIKDVYSNQ
AtLEC2 : MMLNLKNNVQISD----KKDSYQQSTFDNKK-------LRVLCEKELKNSDVGSLGRIVLPKRDAEANLPKLSDKEGIVVQMRDVFSMQ
AtFUS3 : PPISHVPTPLPAR----KIDPRK---------------LRFLFQKELKNSDVSSLRRMILPKKAAEAHLPALECKEGIPIRMEDLDGFH
AtABI3 : PPVMETQLPTMDRAGSASAMPRQQVVPDRRQGWKPEKNLRFLLQKVLKQSDVGNLGRIVLPKKEAETHLPELEARDGISLAMEDIGTSR

TcLEC2 : VWTLKYKFWSNNKSRMYVLENTGDFVKQNGLEIGDSLTLYEDESKNLYFSIIKLERIIAANPPSNHQHSDNENNYYSNNDHHHMYLPFT
AtLEC2 : SWSFKYKEWSNNKSRMYVLENTGEFVKQNGAEIGDFLTIYEDESKNLYF---------AMNGNSGKQNEGRENESRERNHYEEAMLDY-
AtFUS3 : VWTFKYRYWPNNNSRMYVLENTGDFVNAHGLQLGDFIMVYQDLYSNNYV----------IQARKASEEEEVDVINLEEDD------VYT
AtABI3 : VWNMRYREWPNNKSRMYLLENTGDFVKTNGLQEGDFIVIYSDVKCGKYL----------IRGVKVRQPSGQKPEAP----------PSS

TcLEC2 : CQSRDEEETSLELLIEQLKHKEQQETNDLMSLPMIAAY----SHRLPEEAKSFLSDADGVASMETYTPIAATTLQTSSTLLRGKAKSVD
AtLEC2 : -IPRDEEEASIAMLIGNLNDHYPI-PNDLMDLTTDLQHHQATSSSMPPEDHAYVGSSD-------------------------------
AtFUS3 : NLTRIENTVVNDLLLQDFNHHNNNNNNNSNSNSNKCSYYYPVIDDVTINTESFVYDTTALTSNDTPLDFLGGHTTTTNNYYSKFG----
AtABI3 : AATKRQNKSQ-----RNINNNSPS-ANVVVASPTS------------------------------------------------------

TcLEC2 : DFQLNNFDDCYGGLDMLPDVNHYNESL-
AtLEC2 : -----------------DQVSFNDEEWW
AtFUS3 : ---------TFDGLGSVENISLDDEY--
AtABI3 : -----------------QTVK-------
```
