# Supplementary material for: The Theobroma cacao B3 domain transcription factor TcLEC2 plays a duel role in control of embryo development and maturation
Source: BMC Plant Biol. 2014 Apr 24;14:106. doi: 10.1186/1471-2229-14-106 (PMC4021495; doi:10.1186/1471-2229-14-106)

Additional file 3. **Ectopic overexpression of control vector (pGH00.0126) and E12Ω::TcLEC2 in cacao attached leaf transient assay.** Fluorescent micrographs of GFP expression (visualization marker) in leaves were captured three days after transformation (Bars = 0.4mm). **A.** GFP fluorescence image of cacao stage C leaves transformed with control vector. **B.** GFP fluorescence image of cacao stage C leaves transformed with E12Ω::TcLEC2.

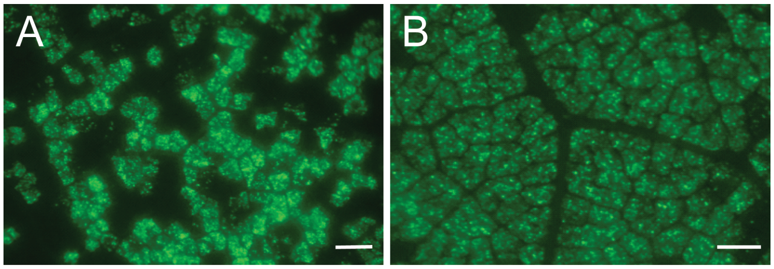

Supplement: Additional file 3 — Ectopic overexpression of control vector (pGH00.0126) and E12Ω::TcLEC2 in cacao attached leaf transient assay. Fluorescent micrographs of GFP expression (visualization marker) in leaves were captured three days after transformation (Bars = 0.4mm). A. GFP fluorescence image of cacao stage C leaves transformed with control vector. B. GFP fluorescence image of cacao stage C leaves transformed with E12Ω::TcLEC2. [file 1471-2229-14-106-S3.pdf]
