# Supplementary material for: The Theobroma cacao B3 domain transcription factor TcLEC2 plays a duel role in control of embryo development and maturation
Source: BMC Plant Biol. 2014 Apr 24;14:106. doi: 10.1186/1471-2229-14-106 (PMC4021495; doi:10.1186/1471-2229-14-106)

Additional file 4. Vector map of E12Ω::TcLEC2. Location of the TcLEC2 and GFP transgenes are indicated as are the NPTII selectable marker genes, and the location of all plant promoter and terminator elements. The control vector plasmid (pGH00.0126, GenBank: KF018690.1) is identical but lacks the E12Ω-TcLEC2-35S Terminator transgene segment.

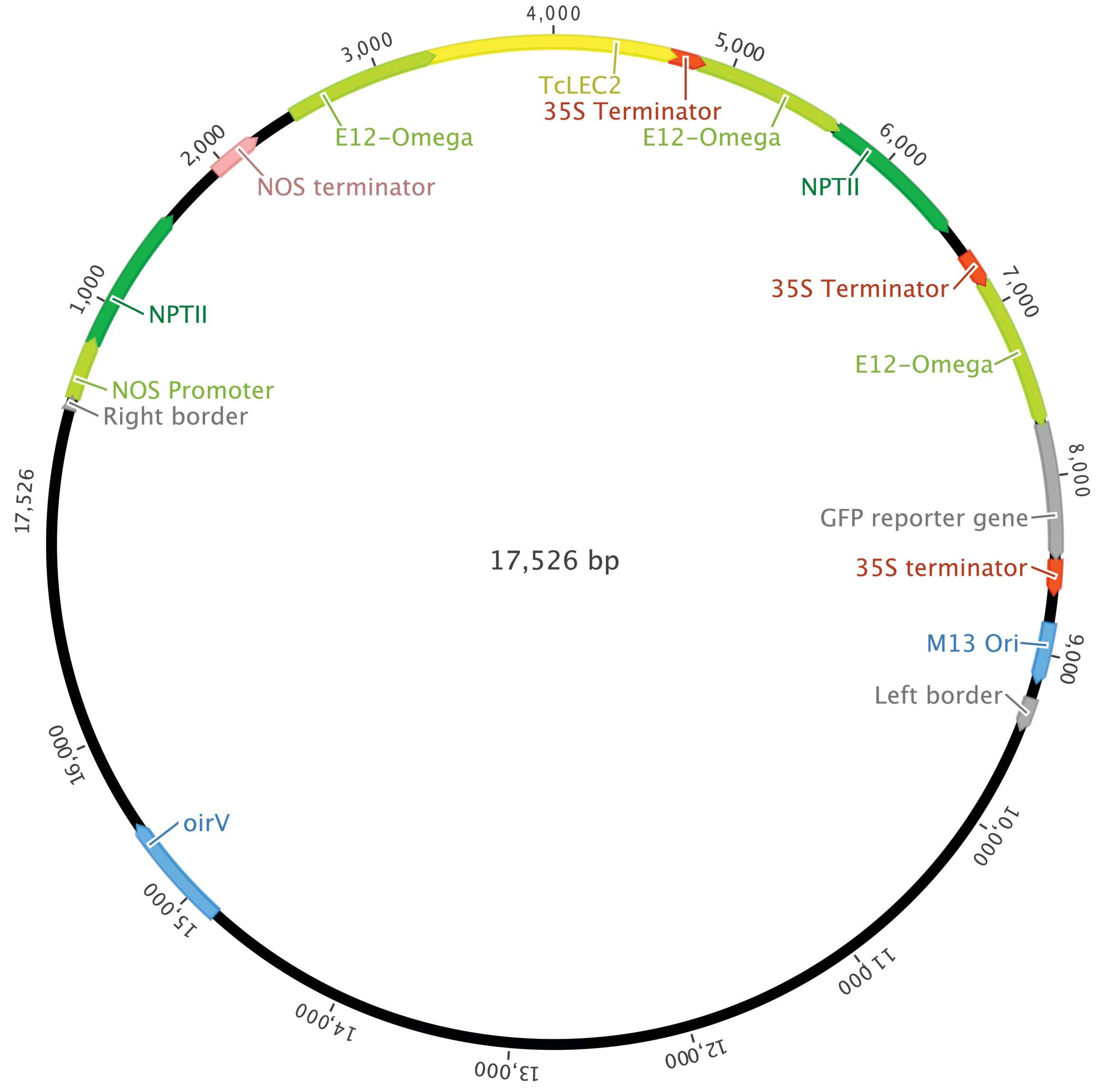

Supplement: Additional file 4 — Vector map of E12Ω::TcLEC2. Location of the TcLEC2 and GFP transgenes are indicated as are the NPTII selectable marker genes, and the location of all plant promoter and terminator elements. The control vector plasmid (pGH00.0126, GenBank: KF018690.1) is identical but lacks the E12Ω-TcLEC2-35S Terminator transgene segment. [file 1471-2229-14-106-S4.pdf]
