# Supplementary material for: The Theobroma cacao B3 domain transcription factor TcLEC2 plays a duel role in control of embryo development and maturation
Source: BMC Plant Biol. 2014 Apr 24;14:106. doi: 10.1186/1471-2229-14-106 (PMC4021495; doi:10.1186/1471-2229-14-106)

Additional file 5. **Relative transient GFP expression levels of TcLEC2 transformation in SE compared to PSUSCA6.**

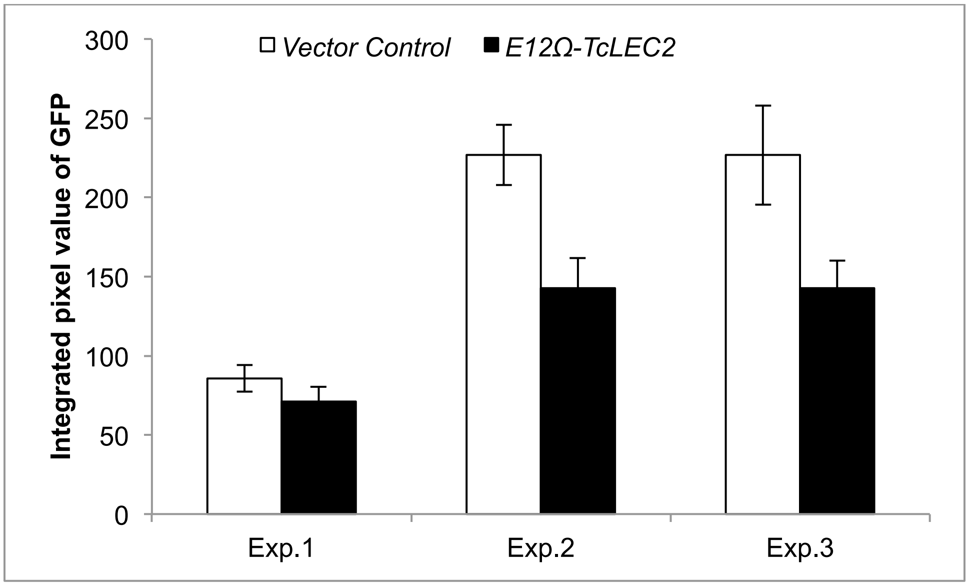

Supplement: Additional file 5 — Relative transient GFP expression levels of TcLEC2 transformation in SE compared to PSUSCA6. [file 1471-2229-14-106-S5.pdf]
