# Supplementary material for: The Theobroma cacao B3 domain transcription factor TcLEC2 plays a duel role in control of embryo development and maturation
Source: BMC Plant Biol. 2014 Apr 24;14:106. doi: 10.1186/1471-2229-14-106 (PMC4021495; doi:10.1186/1471-2229-14-106)

Additional file 6. **Comparison of average of total number of non-transgenic embryo produced per cotyledonary explant.** Sixteen pieces of cotyeldonary explants were placed on each media plate. Three or four plates (taken as biological replicates) were used for transient transformation of control vector or E12Ω::TcLEC2 in each transformation trial (n=3 or 4, mean ± SE). **A.** Transformation trial 1 (n=3). **B.** Transformation trial 2 (n=4). **C.** Transformation trial 3 (n=4).

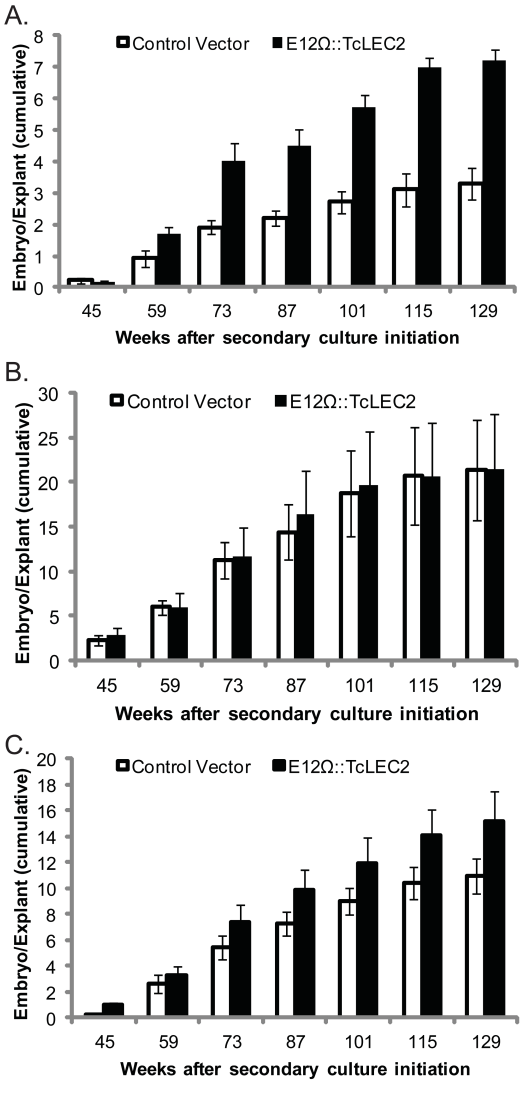

Supplement: Additional file 6 — Comparison of average of total number of non-transgenic embryo produced per cotyledonary explant. Sixteen pieces of cotyeldonary explants were placed on each media plate. Three or four plates (taken as biological replicates) were used for transient transformation of control vector or E12Ω::TcLEC2 in each transformation trial (n=3 or 4, mean ± SE). A. Transformation trial 1 (n=3). B. Transformation trial 2 (n=4). C. Transformation trial 3 (n=4). [file 1471-2229-14-106-S6.pdf]
