# Supplementary material for: The Theobroma cacao B3 domain transcription factor TcLEC2 plays a duel role in control of embryo development and maturation
Source: BMC Plant Biol. 2014 Apr 24;14:106. doi: 10.1186/1471-2229-14-106 (PMC4021495; doi:10.1186/1471-2229-14-106)

Additional file 7. **Overexpression of control vector and E12Ω::TcLEC2 in cacao zygotic embryo transient assay.** Fluorescent micrographs of GFP expression (visualization marker) in leaves were captured five days after transformation (Bars = 2mm). **A&B.** IZE transformed with control vector with white light and GFP fluorescence imaging. **C&D.** IZE transformed with E12Ω::TcLEC2 with white light and GFP fluorescence imaging.

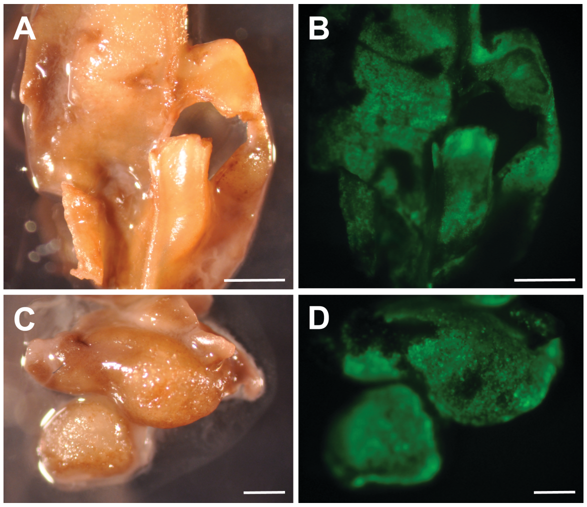

Supplement: Additional file 7 — Overexpression of control vector and E12Ω::TcLEC2 in cacao zygotic embryo transient assay. Fluorescent micrographs of GFP expression (visualization marker) in leaves were captured five days after transformation (Bars = 2mm). A &B. IZE transformed with control vector with white light and GFP fluorescence imaging. C &D. IZE transformed with E12Ω::TcLEC2 with white light and GFP fluorescence imaging. [file 1471-2229-14-106-S7.pdf]
