# Supplementary material for: The Theobroma cacao B3 domain transcription factor TcLEC2 plays a duel role in control of embryo development and maturation
Source: BMC Plant Biol. 2014 Apr 24;14:106. doi: 10.1186/1471-2229-14-106 (PMC4021495; doi:10.1186/1471-2229-14-106)
Supplement: Additional file 8 — Expression levels of genes that are not significantly affected by transient overexpression of TcLEC2 in cacao IZE compared to control vector (n=3, mean ± SE, significant levels were determined by t-test). The gene encoding TcWRI1 was also measured but no expression was detected. [file 1471-2229-14-106-S8.pdf]

Additional file 8. **List of fatty acid biosynthesis related genes in cacao.** The expression of these genes were compared in cacao IZE transiently overexpressing control vector and E12Ω::TcLEC2.

Gene	Tc_ID
TcKASII	*Tc09g006480*
TcFAB2.2	*Tc04g017510*
TcFAB2.7	*Tc08g012550*
TcFAD2.1	*Tc05g018800*
TcFAD2.2	*Tc01g015280*
TcFAD6	*Tc09g002840*
TcFAD7/8	*Tc05g002310*
TcFatA	*Tc01g022130*
TcFatB_1	*Tc09g010360*
TcFatB_2	*Tc01g022130*
TcFatB_5	*Tc03g015170*
TcDGAT1.1	*Tc09g007600*
TcDGAT2	*Tc01g000140*
TcPDAT1	*Tc09g029110*
