# Supplementary material for: The Theobroma cacao B3 domain transcription factor TcLEC2 plays a duel role in control of embryo development and maturation
Source: BMC Plant Biol. 2014 Apr 24;14:106. doi: 10.1186/1471-2229-14-106 (PMC4021495; doi:10.1186/1471-2229-14-106)

Additional file 9. **Expression levels of genes that are not significantly affected by transient overexpression of *TcLEC2* in cacao IZE compared to control vector** (n=3, mean ± SE, significant levels were determined by t-test). The gene encoding TcWRI1 was also measured but no expression was detected.

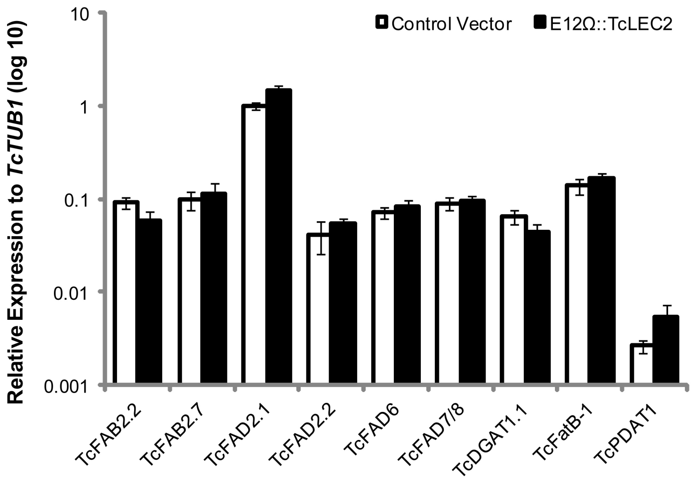

Supplement: Additional file 9 — List of fatty acid biosynthesis related genes in cacao. The expression of these genes were compared in cacao IZE transiently overexpressing control vector and E12Ω::TcLEC2. [file 1471-2229-14-106-S9.pdf]
